# Vitamin A and E status across the spectrum of Hashimoto’s thyroiditis in women: associations with autoimmunity and thyroid function

**DOI:** 10.3389/fnut.2026.1701808

**Published:** 2026-02-19

**Authors:** Qijun Liang, Dongcai Li, Junwei Lin, Zhenhong Qi

**Affiliations:** 1 Health Management Center, The Eighth Clinical Medical College of Guangzhou University of Chinese Medicine, Foshan, Guangdong, China; 2 Foshan Hospital of Traditional Chinese Medicine, Foshan, Guangdong, China

**Keywords:** Hashimoto’s thyroiditis, autoimmunity, thyroid function, women, vitamin A, vitamin E

## Abstract

**Introduction:**

The status of fat-soluble vitamins (A, D, E) across the spectrum ofHashimoto’s thyroiditis (HT), particularly regarding sex-specific patterns, remainsincompletely characterized.

**Methods:**

This cross-sectional study aimed to evaluate the associations of these vitamins with thyroid autoimmunity and function. We analyzed 196 adults, focusing on the 136 women stratified into healthy controls (HC), euthyroid HT (E-HT), and dysfunctional HT (HT-dys).

**Results:**

In women, serum vitamin A levels were significantly lower in both the E-HT and HT-dys groups compared to the HC group. Lipid-normalized vitamin E (*α*-tocopherol/[total cholesterol+triglycerides], αT/[TCH+TG]) was significantly reduced in the HT-dys group compared to both the HC and E-HT groups. Vitamin A levels showed an inverse association with thyroglobulin antibody (TgAb), while αT/(TCH+TG) was inversely associated with both thyroid peroxidase antibody (TPOAb) and TgAb. Among women with HT, αT/(TCH+TG) was inversely correlated with thyroid-stimulating hormone (TSH) and positively correlated with free thyroxine (FT4). An exploratory analysis indicated that this association between vitamin E and thyroid function was largely independent of TPOAb levels.

**Conclusion:**

This exploratory study identifies distinct associations of vitamins A and E with disease activity in women with HT, highlighting the need for longitudinal and interventional studies to further explore these relationships.

## 1 Introduction

Hashimoto’s thyroiditis (HT), the most prevalent autoimmune thyroid disorder, is a leading cause of hypothyroidism and demonstrates a marked female predominance (1, 2). The pathogenesis of HT involves immune-mediated thyroid destruction, in which oxidative stress acts as a key amplifier of tissue damage (3–6). Given this pathophysiology, fat-soluble vitamins have garnered interest due to their immunomodulatory and antioxidant properties.

Critical evidence gaps remain, however. Vitamin A (retinol) is essential for immune regulation, primarily through retinoid receptor-mediated T-cell modulation (7–9). Deficiency in vitamin A is known to impair thyroid hormone synthesis (10–12), yet direct assessments of vitamin A status in patients with HT are limited. Vitamin D exerts potent immunomodulatory effects (13–15). Although some supplementation trials have shown promise in reducing thyroid autoantibodies (16, 17), observational studies on the association between serum vitamin D and HT have yielded inconsistent results (18–22), leaving its role unclear. Vitamin E, particularly *α*-tocopherol (αT), is a key lipid-soluble antioxidant that protects against peroxidation and modulates immune function (23, 24). Although epidemiological links have been reported (25), high-quality evidence quantifying vitamin E status in HT using precise methods such as liquid chromatography–tandem mass spectrometry (LC–MS/MS) remains scarce, and the important step of lipid normalization is frequently overlooked.

These gaps point to two unresolved questions. First, the sex-specific patterns of these vitamins across the spectrum of HT, from euthyroidism to overt dysfunction, remain poorly characterized, despite the strong female predominance of the disease. Second, there is a notable lack of comprehensive studies that concurrently evaluate all three vitamins with rigorous methodology, particularly with respect to LC–MS/MS and lipid normalization for vitamin E.

Therefore, we conducted a cross-sectional study to: (1) characterize the sex-specific status of vitamins A, D, and E across HT disease categories using high-precision LC–MS/MS and lipid-normalized vitamin E levels; (2) examine the associations of these vitamins with thyroid autoimmunity (thyroid peroxidase antibody [TPOAb] and thyroglobulin antibody [TgAb]) and thyroid function (thyroid-stimulating hormone [TSH] and free thyroxine [FT4]); and (3) explore the complex relationships and potential interplay among these vitamins, autoantibodies, and thyroid function. By focusing on sex-stratified analyses and methodologically robust assessments, this study aims to provide novel insights into the potential role of these nutritional factors in HT.

## 2 Materials and methods

### 2.1 Study population

The study enrolled Chinese adults aged 18–75 years who underwent health check-ups at the Health Management Center of Foshan Hospital of Traditional Chinese Medicine between January and March 2025. All participants completed a standardized assessment, which included biochemical tests, measurements of serum vitamins (A, D, and E), thyroid function tests (TSH and FT4), thyroid autoantibody assays (TPOAb and TgAb), and thyroid ultrasonography. Exclusion criteria were as follows: (a) other thyroid diseases, such as Graves’ disease or subacute thyroiditis; (b) current or previous use of thyroid medications (e.g., levothyroxine, methimazole or propylthiouracil) or a history of thyroid surgery or radioactive iodine therapy; (c) presence of other endocrine disorders or use of hormone therapy; (d) diagnosis of malignancy; and (e) pregnancy or lactation.

Data collection followed the standardized protocols of the health check-up program. Consequently, information on certain potential confounding variables, including detailed dietary intake, use of vitamin supplements, and sunlight exposure, was not routinely collected and was therefore unavailable for adjustment.

The study was conducted in accordance with the Declaration of Helsinki, and was approved by the Ethics Committee of Foshan Hospital of Traditional Chinese Medicine.

### 2.2 Data collection

#### 2.2.1 Anthropometry

Height and weight were measured with participants wearing light clothing and without shoes. Body mass index (BMI) was calculated as weight in kilograms divided by the square of height in meters (kg/m^2^). After a 10-min rest, systolic and diastolic blood pressure (SBP and DBP) were measured three times on the right arm using an appropriately sized cuff; the average of the three readings was recorded.

#### 2.2.2 Biochemical analyses

Fasting venous blood samples were collected after an 8- to 12-h overnight fast to minimize dietary influences on metabolic parameters and vitamin levels. All samples were drawn between 7:00 and 9:00 a.m. to reduce diurnal variation. Fasting plasma glucose (FPG) was measured using the hexokinase/G-6-PDH method. Enzymatic methods were used to assess triglycerides (TG) and total cholesterol (TC) with glycerol-3-phosphate kinase and cholesterol esterase-cholesterol oxidase, respectively. Low-density lipoprotein cholesterol (LDL-C) and high-density lipoprotein cholesterol (HDL-C) were measured by direct methods. Uric acid (UA) was determined by uricase method. Creatinine (Cr) was measured via the sarcosine oxidase method, and urea was measured using urease rate method. Alanine aminotransferase (ALT), aspartate aminotransferase (AST), and *γ*-glutamyl transferase (GGT) levels were measured by kinetic assays.

#### 2.2.3 Thyroid function and autoantibodies

Serum FT4 and TSH levels were quantified using chemiluminescence immunoassays on a Siemens ADVIA Centaur XP analyzer. Serum concentrations of TPOAb and TgAb were determined by electrochemiluminescence immunoassays on a Roche Cobas 6,000 analyzer.

#### 2.2.4 Vitamin analysis

Serum concentrations of vitamin A (retinol), vitamin D (25-hydroxyvitamin D, 25[OH]D), and vitamin E (*α*-tocopherol, α-T) were quantified by LC–MS/MS. Analyses were performed using an AB SCIEX 5500+QTrap mass spectrometer coupled to a Shimadzu Nevera X2 LC system and commercially available isotope-dilution mass spectrometry (IDMS) kits (Chromsystems Instruments & Chemicals GmbH, Gräfelfing, Germany). Intra- and inter-assay coefficients of variation were both below 10%. Given the strong lipid dependence of tocopherol transport, *α*-T concentrations were normalized to total lipids using the ratio α-tocopherol/(total cholesterol+triglycerides) (αT/[TCH+TG]). The normalization minimizes confounding from interindividual variations in lipid metabolism and better reflects tissue-available vitamin E status (26, 27).

#### 2.2.5 Thyroid ultrasonography

Thyroid ultrasound examinations were performed by senior sonographers, each with a minimum of 10 years of thyroid imaging experience, using a high-resolution B-mode ultrasound system (MyLab Class C, Esaote, Italy). Participants were positioned supine with the necks slightly extended.

### 2.3 Definition and grouping

HT was diagnosed based on the presence of thyroid autoantibody positivity (defined as TPOAb ≥ 34 IU/mL and/or TgAb ≥ 115 IU/mL) and characteristic heterogeneous echotexture with diffuse or patchy hypoechogenicity on thyroid ultrasonography (28).

Participants were stratified into three groups according to their autoantibody status and thyroid function: healthy controls (HC) (thyroid autoantibody negative [TPOAb < 34 IU/mL and TgAb < 115 IU/mL] with normal thyroid function [TSH 0.6–4.8 mIU/L, FT4 12–20.2 pmol/L]), euthyroid HT (E-HT) (positive for TPOAb and/or TgAb, with maintained euthyroidism), and dysfunctional HT (HT-dys) (positive for TPOAb and/or TgAb, with biochemically confirmed subclinical [TSH > 4.8 mIU/L and FT4 12–20.2 pmol/L] or overt hypothyroidism [TSH > 4.8 mIU/L and FT4 < 12 pmol/L]).

Serum 25(OH)D < 20 ng/mL was defined as vitamin D deficiency, in according with the Endocrine Society guidelines (29).

### 2.4 Statistical analysis

Statistical analyses were performed using SPSS version 27.0 (IBM Corp., Armonk, NY, USA). Continuous variables are presented as mean ± standard deviation (SD) and categorical variables as number (%). Intergroup differences were assessed using *t*-tests, *χ*^2^ tests, or one-way ANOVA with LSD post-hoc tests, as appropriate.

For the female cohort, multivariable linear regression (enter method) was used to identify determinants of thyroid autoimmunity and function. Covariates were selected *a priori* based on biological plausibility and clinical relevance, including demographic factors (age), anthropometric measures (BMI, SBP and DBP), metabolic parameters (FPG, lipid profiles, UA, liver and renal function markers), and thyroid-specific parameters (thyroid function tests and autoantibodies). Multicollinearity among candidate variables was assessed using the variance inflation factor (VIF); no variables exhibited significant collinearity (all VIFs < 5), and all were retained. The analysis was conducted as a complete-case analysis owing to the absence of missing data for key variables. To test for effect modification by sex, vitamin × sex interaction terms were included in the regression models.

An exploratory model-based analysis was performed to examine whether the association between lipid-normalized vitamin E (αT/[TCH+TG]) and thyroid function (TSH/FT4) operated through a pathway shared with or independent of TPOAb levels. It is critical to emphasize that this cross-sectional analysis does not imply causality or temporal sequence.

Given the exploratory nature of this study and the multiple statistical tests performed, we did not adjust for multiple comparisons (e.g., Bonferroni or false discovery rate) to avoid unduly increasing the risk of Type II errors (false negatives) and to allow for hypotheses generation. Consequently, all *p*-values, especially those near the conventional threshold (*p* < 0.05), should be interpreted with caution as hypotheses-generating rather than confirming robust associations. Statistical significance was set at *p* < 0.05 (two-sided).

The sample size was determined by feasibility and recruitment during the study period. No formal *a priori* power calculation was performed. Therefore, the analyses, particularly sex-stratified and subgroup comparisons, may be underpowered to detect modest effect sizes, and the risk of Type II errors should be considered.

Owing to the very small sample size of the male HT-dys group (*n* = 4), which precludes valid statistical inference, data for the male cohort are presented as descriptive only. No *p*-values are reported for male comparisons, and these results should be interpreted as hypothesis-generating.

## 3 Results

The study included 196 participants (136 women, 60 men) with a mean age of 46.3 ± 10.9 years. As summarized in Table 1, men exhibited significantly higher values than women on anthropometrics measures (height, weight, BMI), blood pressure, various metabolic markers (TG, UA, Cr, Urea), liver enzymes (ALT, GGT), thyroid hormones (FT4), and vitamin A levels (625.37 ± 257.79 ng/mL vs. 431.40 ± 116.78 ng/mL, *p* < 0.001). Conversely, women had significantly higher HDL-C, elevated TPOAb titers, a greater prevalence of HT (60.29% vs. 41.67%, *p* = 0.016) and a higher prevalence of vitamin D deficiency (26.47% vs. 5.00%, *p* < 0.001) compared to men.

**Table 1:** Sex-specific baseline characteristics of the study population (*n* = 196).

Parameter	Male (*n* = 60)	Women (*n* = 136)	*p*
Age (years)	48.55 ± 10.44	45.26 ± 11.03	0.052
Height (cm)	170.53 ± 5.38	158.05 ± 5.24	<0.001
Weight (kg)	73.84 ± 9.35	57.45 ± 8.01	<0.001
BMI (kg/m^2^)	25.37 ± 2.77	23.00 ± 2.90	<0.001
SBP (mmHg)	129.93 ± 11.54	118.96 ± 13.23	<0.001
DBP (mmHg)	82.23 ± 9.42	73.11 ± 9.08	<0.001
FPG (mmol/L)	5.32 ± 0.96	5.11 ± 1.09	0.202
TG (mmol/L)	2.36 ± 3.84	1.28 ± 0.59	0.002
TCH (mmol/L)	5.14 ± 1.25	5.43 ± 1.06	0.102
LDL-C (mmol/L)	3.35 ± 0.99	3.40 ± 0.79	0.730
HDL-C (mmol/L)	1.17 ± 0.23	1.47 ± 0.30	<0.001
UA (μmol/L)	425.96 ± 92.89	315.03 ± 66.16	<0.001
Cr (μmol/L)	77.83 ± 14.31	60.96 ± 10.47	<0.001
Urea (mmol/L)	4.86 ± 0.96	4.38 ± 1.14	0.005
ALT (U/L)	24.87 ± 9.13	18.92 ± 13.20	0.002
AST (U/L)	22.63 ± 6.30	20.61 ± 7.76	0.077
GGT (U/L)	29.16 ± 11.07	19.79 ± 11.97	<0.001
FT4 (pmol/L)	16.83 ± 2.95	15.64 ± 3.20	0.015
TSH (mIU/L)	3.47 ± 6.40	4.93 ± 12.60	0.393
HT	25 (41.67%)	82 (60.29%)	0.016
TPOAb (IU/mL)	75.88 ± 142.24	126.36 ± 169.39	0.033
TgAb (IU/mL)	226.69 ± 575.19	326.62 ± 709.15	0.338
Vitamin A (ng/mL)	625.37 ± 257.79	431.40 ± 116.78	<0.001
25(OH)D (ng/mL)	25.53 ± 7.03	25.43 ± 10.19	0.937
Vitamin D deficiency	3 (5.00%)	36 (26.47%)	<0.001
αT (ng/mL)	10137.49 ± 3882.73	10114.23 ± 3343.67	0.968
αT/(TCH+TG) (μg/mmol)	1407.62 ± 291.90	1508.22 ± 391.66	0.076

Data presented as mean ± SD or *n* (%). *p*-values derived from independent *t*-tests or χ^2^ tests. BMI, body mass index; SBP, systolic blood pressure; DBP, diastolic blood pressure; FPG, fasting plasma glucose; TG, triglycerides; TCH, total cholesterol; LDL-C, low-density lipoprotein cholesterol; HDL-C, high-density lipoprotein cholesterol; UA, uric acid; Cr, creatinine; ALT, alanine transaminase; AST, aspartate aminotransferase; GGT, γ-glutamyl transpeptidase; Cr, creatinine; FT4, free thyroxine; TSH, thyroid-simulating hormone; HT, Hashimoto’s thyroiditis; TPOAb, thyroid peroxidase antibody; TgAb, anti-thyroglobulin antibody; 25(OH)D, 25-hydroxyvitamin D; αT, α-tocopherol; αT/(TCH+TG), α-tocopherol/(total cholesterol+triglycerides).

Analysis of the female cohort revealed significant differences in vitamin status across the study groups (Table 2). Serum vitamin A concentrations were significantly lower in both the E-HT and HT-dys groups compared to the HC group. αT/(TCH+TG) was significantly reduced in the HT-dys group (1188.95 ± 305.70 μg/mmol) compared to both the HC group (1606.03 ± 357.18 μg/mmol; 26% decrease, *p* < 0.05) and the E-HT group (1496.28 ± 401.09 μg/mmol; 21% decrease, *p* < 0.05). In contrast, no significant intergroup differences were observed in serum vitamin D levels or in the prevalence of vitamin D deficiency.

**Table 2:** Vitamin status and clinical characteristics of the female cohort across the spectrum of Hashimoto’s thyroiditis (*n* = 136).

Parameter	HC	E-HT	HT-dys	*p*
*n*	54	68	14	
Age (years)	44.98 ± 12.49	44.68 ± 9.55	49.21 ± 11.79	0.366
Height (cm)	158.66 ± 4.53	158.05 ± 5.74	155.65 ± 4.88	0.159
Weight (kg)	58.05 ± 6.27	57.31 ± 9.13	55.79 ± 8.50	0.643
BMI (kg/m^2^)	23.10 ± 2.26	22.92 ± 3.40	22.99 ± 2.63	0.943
SBP (mmHg)	119.33 ± 14.79	117.81 ± 11.80	123.14 ± 13.49	0.379
DBP (mmHg)	73.11 ± 9.08	71.66 ± 8.44	73.86 ± 9.49	0.549
FPG (mmol/L)	5.19 ± 1.61	5.01 ± 0.46	5.26 ± 0.79	0.599
TG (mmol/L)	1.20 ± 0.49	1.29 ± 0.65	1.59 ± 0.63	0.092
TCH (mmol/L)	5.38 ± 1.09	5.51 ± 0.93	5.23 ± 1.51	0.585
LDL-C (mmol/L)	3.42 ± 0.85	3.41 ± 0.67	3.25 ± 1.10	0.777
HDL-C (mmol/L)	1.44 ± 0.27	1.50 ± 0.29	1.43 ± 0.45	0.492
UA (μmol/L)	317.10 ± 76.32	308.11 ± 56.65	340.71 ± 64.73	0.235
Cr (μmol/L)	62.71 ± 12.56	59.90 ± 7.96	60.96 ± 10.47	0.283
Urea (mmol/L)	4.49 ± 1.18	4.30 ± 1.13	4.30 ± 1.00	0.639
ALT (U/L)	18.15 ± 7.84	18.48 ± 16.76	24.00 ± 8.90	0.314
AST (U/L)	20.40 ± 7.75	20.18 ± 8.04	23.49 ± 6.01	0.339
GGT (U/L)	19.67 ± 12.82	19.72 ± 11.34	20.57 ± 12.43	0.967
FT4 (pmol/L)	16.36 ± 3.04	15.82 ± 2.21	11.98 ± 5.21^bc^	<0.001
TSH (mIU/L)	2.30 ± 2.20	2.22 ± 1.25	28.23 ± 31.14^bc^	<0.001
TPOAb (IU/mL)	12.92 ± 4.18	179.68 ± 168.29^a^	304.93 ± 221.98^bc^	<0.001
TgAb (IU/mL)	19.47 ± 12.23	383.46 ± 616.99^a^	1235.28 ± 1400.69^bc^	<0.001
Vitamin A (ng/mL)	461.72 ± 118.95	419.06 ± 113.39^a^	374.39 ± 97.75^b^	0.020
25(OH)D (ng/mL)	27.04 ± 12.56	23.81 ± 7.90	27.06 ± 9.35	0.183
Vitamin D deficiency	12 (22.22%)	22 (32.36%)	2 (14.29%)	0.249
αT (ng/mL)	10562.37 ± 3104.73	10111.62 ± 3382.02	8398.36 ± 3721.27^b^	0.097
αT/(TCH+TG) (μg/mmol)	1606.03 ± 357.18	1496.28 ± 401.09	1188.95 ± 305.70^bc^	0.001

Data presented as mean ± SD or *n* (%). *p*-values derived from independent ANOVA or χ^2^ tests. HT, Hashimoto’s thyroiditis; HC, healthy controls; E-HT, euthyroid HT; HT-dys, dysfunctional HT; BMI, body mass index; SBP, systolic blood pressure; DBP, diastolic blood pressure; FPG, fasting plasma glucose; TG, triglycerides; TCH, total cholesterol; LDL-C, low-density lipoprotein cholesterol; HDL-C, high-density lipoprotein cholesterol; UA, uric acid; Cr, creatinine; ALT, alanine transaminase; AST, aspartate aminotransferase; GGT, γ-glutamyl transpeptidase; Cr, creatinine; FT4, free thyroxine; TSH, thyroid-simulating hormone; TPOAb, thyroid peroxidase antibody; TgAb, anti-thyroglobulin antibody; 25(OH)D, 25-hydroxyvitamin D; αT, α-tocopherol; αT/(TCH+TG), α-tocopherol/(total cholesterol+triglycerides).

^a^Group E-HT vs. group HC, *p* < 0.05.

^b^Group HT-dys vs. group HC, *p* < 0.05.

^c^Group HT-dys vs. group E-HT, *p* < 0.05.

Given the very limited sample size, particularly in the male HT-dys group (*n* = 4), data for the male cohort are presented in Supplementary Table S1 solely for descriptive purposes. No statistical comparisons between male subgroups were performed or reported, as such comparisons would be statistically unreliable.

Multivariable linear regression analysis revealed distinct determinants of thyroid autoimmunity in women (Table 3). TPOAb levels showed positive associations with both TSH (*β* = 3.917, 95% CI [1.162, 6.672], *p* = 0.006) and TgAb (*β* = 0.093, 95% CI [0.055, 0.131], *p* < 0.001), but an inverse association with αT/(TCH+TG) (*β* = −0.078, 95% CI [−0.152, −0.005], *p* = 0.038). It should be noted that this *p*-value for the latter association is marginal and should be considered exploratory in the context of multiple testing. Similarly, TgAb levels were positively associated with TPOAb (*β* = 1.878, 95% CI [1.109, 2.647], *p* < 0.001) and inversely associated with both vitamin A (*β* = −1.531, 95% CI [−2.679, −0.383], *p* = 0.009) and αT/(TCH+TG) (*β* = −0.369, 95% CI [−0.670, −0.067], *p* = 0.015).

**Table 3:** Determinants of thyroid autoantibodies in women: results from multivariable linear regression analysis (*n* = 136).

Outcome	Predictor	*β* (95% CI)	*p*
TPOAb (IU/mL)	Age (years)	−1.496 (−4.252, 1.261)	0.285
	Height (cm)	−2.555 (−8.568, 3.459)	0.402
	Weight (kg)	1.896 (−2.354, 6.147)	0.379
	BMI (kg/m^2^)	−8.096 (−17.643, 1.451)	0.096
	SBP (mmHg)	−0.211 (−3.435, 3.013)	0.897
	DBP (mmHg)	0.907 (−3.656, 5.470)	0.694
	FPG (mmol/L)	−10.740 (−34.498, 13.018)	0.372
	TG (mmol/L)	36.062 (−23.415, 95.539)	0.232
	TCH (mmol/L)	63.699 (−11.648, 139.046)	0.097
	LDL-C (mmol/L)	−70.774 (−153.312, 11.763)	0.092
	HDL-C (mmol/L)	25.432 (−92.663, 143.526)	0.670
	UA (μmol/L)	−0.078 (−0.531, 0.374)	0.732
	Cr (μmol/L)	0.722 (−2.008, 3.452)	0.601
	Urea (mmol/L)	7.885 (−16.450, 32.221)	0.522
	ALT (U/L)	−0.529 (−4.265, 3.207)	0.780
	AST (U/L)	0.860 (−4.905, 6.624)	0.768
	GGT (U/L)	−0.503 (−3.109, 2.104)	0.703
	FT4 (pmol/L)	−20.798 (−68.162, 26.566)	0.386
	TSH (mIU/L)	3.917 (1.162, 6.672)	0.006
	TgAb (IU/mL)	0.093 (0.055, 0.131)	<0.001
	Vitamin A (ng/mL)	0.024 (−0.239, 0.287)	0.856
	25(OH)D (ng/mL)	−0.909 (−3.520, 1.702)	0.492
	αT (ng/mL)	−0.071 (−0.142, −0.001)	0.054
	αT/(TCH+TG) (μg/mmol)	−0.078 (−0.152, −0.005)	0.038
TgAb (IU/mL)	Age (years)	12.008 (−0.253, 24.269)	0.055
	Height (cm)	−9.577 (−36.661, 17.506)	0.485
	Weight (kg)	1.873 (−17.315, 21.061)	0.847
	BMI (kg/m^2^)	−0.138 (−43.639, 43.363)	0.995
	SBP (mmHg)	−3.490 (−17.985, 11.005)	0.634
	DBP (mmHg)	3.849 (−16.685, 24.383)	0.711
	FPG (mmol/L)	−25.212 (−132.393, 81.969)	0.642
	TG (mmol/L)	22.617 (−36.735, 81.969)	0.090
	TCH (mmol/L)	−220.418 (−561.192, 120.357)	0.203
	LDL-C (mmol/L)	275.424 (−97.174, 648.021)	0.146
	HDL-C (mmol/L)	−128.415 (−659.677, 402.847)	0.633
	UA (μmol/L)	0.291 (−1.746, 2.328)	0.777
	Cr (μmol/L)	−5.674 (−17.925, 6.577)	0.361
	Urea (mmol/L)	−37.932 (−147.400, 71.536)	0.494
	ALT (U/L)	1.549 (−15.265, 18.362)	0.855
	AST (U/L)	−10.153 (−36.031, 15.725)	0.439
	GGT (U/L)	−5.685 (−17.371, 6.001)	0.337
	FT4 (pmol/L)	−5.533 (−48.598, 37.533)	0.800
	TSH (mIU/L)	−1.157 (−13.987, 11.673)	0.858
	TPOAb (IU/mL)	1.878 (1.109, 2.647)	<0.001
	Vitamin A (ng/mL)	−1.531 (−2.679, −0.383)	0.009
	25(OH)D (ng/mL)	7.703 (−3.982, 19.388)	0.194
	αT (ng/mL)	−0.200 (−0.536, 0.136)	0.241
	αT/(TCH+TG) (μg/mmol)	−0.369 (−0.670, −0.067)	0.015

BMI, body mass index; SBP, systolic blood pressure; DBP, diastolic blood pressure; FPG, fasting plasma glucose; TG, triglycerides; TCH, total cholesterol; LDL-C, low-density lipoprotein cholesterol; HDL-C, high-density lipoprotein cholesterol; UA, uric acid; Cr, creatinine; ALT, alanine transaminase; AST, aspartate aminotransferase; GGT, γ-glutamyl transpeptidase; Cr, creatinine; FT4, free thyroxine; TSH, thyroid-simulating hormone; TPOAb, thyroid peroxidase antibody; TgAb, anti-thyroglobulin antibody; 25(OH)D, 25-hydroxyvitamin D; αT, α-tocopherol; αT/(TCH+TG), α-tocopherol/(total cholesterol+triglycerides).

Regarding thyroid function in women with HT, TSH levels demonstrated a positive association with TPOAb (*β* = 0.024, 95% CI [0.006, 0.042], *p* = 0.010) but inverse associations with both FT4 (*β* = −3.026, 95% CI [−3.922, −2.130], *p* < 0.001) and αT/(TCH+TG) (*β* = −0.012, 95% CI [−0.020, −0.003], *p* = 0.009). Conversely, FT4 levels were negatively associated with TSH (*β* = −0.147, 95% CI [−0.191, −0.104], *p* < 0.010) and TPOAb (*β* = −0.004, 95% CI [−0.009, −0.001], *p* = 0.047), but positively associated with αT/(TCH+TG) (*β* = 0.004, 95% CI [0.002, 0.006], *p* = 0.005) (Table 4).

**Table 4:** Associations of clinical variables with thyroid function in women with Hashimoto’s thyroiditis: results from multivariable linear regression analysis (*n* = 82).

Outcome	Predictor	*β* (95%CI)	*p*
TSH	Age (years)	0.069 (−0.252, 0.390)	0.670
	Height (cm)	0.592 (−0.126, 1.311)	0.104
	Weight (kg)	−0.273 (−0.719, 0.172)	0.224
	BMI (kg/m^2^)	−0.150 (−1.117, 0.817)	0.757
	SBP (mmHg)	0.193 (−0.146, 0.532)	0.259
	DBP (mmHg)	−0.081 (−0.532, 0.369)	0.719
	FPG (mmol/L)	1.052 (−4.821, 6.924)	0.721
	TG (mmol/L)	2.238 (−7.079, 11.554)	0.632
	TCH (mmol/L)	11.648 (−0.969, 24.264)	0.074
	LDL-C (mmol/L)	10.750 (−0.761, 22.261)	0.066
	HDL-C (mmol/L)	11.767 (−0.257, 23.791)	0.055
	UA (μmol/L)	0.001 (−0.056, 0.057)	0.994
	Cr (μmol/L)	0.244 (−0.144, 0.633)	0.213
	Urea (mmol/L)	−0.406 (−3.428, 2.615)	0.789
	ALT (U/L)	−0.210 (−0.659, 0.239)	0.354
	AST (U/L)	0.615 (−0.237, 1.466)	0.154
	GGT (U/L)	0.224 (−0.098, 0.546)	0.168
	FT4 (pmol/L)	−3.026 (−3.922, −2.130)	<0.001
	TPOAb (IU/mL)	0.024 (0.006, 0.042)	0.010
	TgAb (IU/mL)	0.000 (−0.004, 0.004)	0.996
	Vitamin A (ng/mL)	−0.008 (−0.037, 0.022)	0.603
	25(OH)D (ng/mL)	−0.100 (−0.461, 0.261)	0.581
	αT (ng/mL)	−0.003 (−0.026, 0.021)	0.805
	αT/(TCH+TG) (μg/mmol)	−0.012 (−0.020, −0.003)	0.009
FT4	Age (years)	0.026 (−0.044, 0.097)	0.461
	Height (cm)	0.140 (−0.018, 0.298)	0.081
	Weight (kg)	−0.063 (−0.162, 0.035)	0.200
	BMI (kg/m^2^)	−0.039 (−0.252, 0.174)	0.714
	SBP (mmHg)	0.038 (−0.037, 0.113)	0.319
	DBP (mmHg)	0.006 (−0.093, 0.106)	0.898
	FPG (mmol/L)	0.291 (−1.003, 1.585)	0.655
	TG (mmol/L)	0.055 (−2.003, 2.114)	0.957
	TCH (mmol/L)	−0.788 (−3.452, 1.876)	0.556
	LDL-C (mmol/L)	0.629 (−1.830, 3.087)	0.611
	HDL-C (mmol/L)	−0.172 (−2.911, 2.567)	0.900
	UA (μmol/L)	−0.006 (−0.019, 0.006)	0.310
	Cr (μmol/L)	0.067 (−0.018, 0.152)	0.120
	Urea (mmol/L)	−0.249 (−0.912, 0.415)	0.456
	ALT (U/L)	−0.013 (−0.113, 0.086)	0.790
	AST (U/L)	0.063 (−0.128, 0.253)	0.511
	GGT (U/L)	0.051 (−0.020, 0.121)	0.158
	TSH (mIU/L)	−0.147 (−0.191, −0.104)	<0.001
	TPOAb (IU/mL)	−0.004 (−0.009, −0.001)	0.047
	TgAb (IU/mL)	0.000 (−0.001, 0.001)	0.579
	Vitamin A (ng/mL)	0.001 (−0.005, 0.008)	0.715
	25(OH)D (ng/mL)	−0.037 (−0.116, 0.042)	0.356
	αT (ng/mL)	0.001 (−0.004, 0.006)	0.711
	αT/(TCH+TG) (μg/mmol)	0.004 (0.002, 0.006)	0.005

BMI, body mass index; SBP, systolic blood pressure; DBP, diastolic blood pressure; FPG, fasting plasma glucose; TG, triglycerides; TCH, total cholesterol; LDL-C, low-density lipoprotein cholesterol; HDL-C, high-density lipoprotein cholesterol; UA, uric acid; Cr, creatinine; ALT, alanine transaminase; AST, aspartate aminotransferase; GGT, γ-glutamyl transpeptidase; Cr, creatinine; TSH, thyroid-simulating hormone; FT4, free thyroxine; TPOAb, thyroid peroxidase antibody; TgAb, anti-thyroglobulin antibody; 25(OH)D, 25-hydroxyvitamin D; αT, α-tocopherol; αT/(TCH+TG), α-tocopherol/(total cholesterol+triglycerides).

Interaction analysis indicated a nominally significant interaction between αT/(TCH+TG) and sex in relation to TgAb levels (*p* = 0.049). However, this marginal interaction effect should be interpreted with extreme caution given the number of tests performed and the lack of adjustment for multiple comparisons. No other significant interactions with sex were observed for the relationships between vitamin A and TgAb, or between αT/(TCH+TG) and TPOAb, TSH, or FT4, either in the overall population or the HT-subgroup (all *p* > 0.05).

Finally, an exploratory model-based analysis demonstrated that the association between αT/(TCH+TG) and thyroid function in women with HT was largely independent of TPOAb levels, with the majority of the statistical association not explained by TPOAb (Supplementary Table S2).

## 4 Discussion

### 4.1 Vitamin A

Our study demonstrates significantly lower serum vitamin A levels in women with HT compared to healthy controls (30, 31), extending earlier observations in Caucasian populations (32) to our Chinese cohort. The key novel finding is the significant inverse association between vitamin A and TgAb, in the absence of a direct association with thyroid function parameters. This specific pattern generates the hypothesis that vitamin A’s role in HT may be primarily immunological. The inverse correlation with TgAb is consistent with its established role in immune regulation, particularly in T-cell function and B-cell activity (12, 33, 34). Conversely, the lack of association with thyroid function aligns with some prior studies (32, 35), suggesting that the vitamin’s effects are likely mediated through immunoregulation rather than a direct impact on thyroid hormone synthesis. These findings positions vitamin A status as a potential modulator of the specific autoimmune response against thyroglobulin in HT.

### 4.2 Vitamin E

A key finding of our study is the inverse association of lipid-normalized vitamin E (αT/(TCH+TG)) with both thyroid autoantibodies (TPOAb, TgAb) and thyroid function (TSH, FT4) in women with HT. The association with autoantibodies aligns with the established immunomodulatory role of vitamin E, which includes regulating dendritic cell maturation, macrophage polarization, and T/B-cell function (23, 36, 37). More importantly, our exploratory analysis further revealed that the association between αT/(TCH+TG) and thyroid function was largely independent of TPOAb levels. This hypothesis-generating finding suggests that, beyond immunomodulation, vitamin E may influence thyroid status through more direct pathways. As a potent lipid-soluble antioxidant, vitamin E protects cellular integrity against peroxidation (38, 39). Given that thyroid hormone biosynthesis is an oxidative process dependent on peroxide generation (40), it is plausible that a lower vitamin E status could exacerbate oxidative stress within the thyroid gland, directly impairing thyrocyte function. This hypothesis is supported by experimental evidence indicating that oxidative stress modulates redox-sensitive signaling pathways critical for thyrocyte activity (41). Thus, vitamin E deficiency may contribute to pathophysiology of HT not only by promoting immune dysregulation but also by increasing the gland’s susceptibility to reactive oxygen species (ROS)-mediated damage.

### 4.3 Vitamin D

In contrast to vitamins A and E, our study found no significant association between vitamin D status and HT in the female cohort. This null finding serves as an important methodological comparator, underscoring the specificity of the associations identified for vitamins A and E, and indicating that they are not a generic feature of all fat-soluble vitamins. While some studies have reported an association between vitamin D and HT (42–44), others have not (20, 21), highlighting the persistent inconsistency in this area. Our results, obtained with a robust methodological framework, suggest that in this specific population, vitamin D status may not be a primary cross-sectional correlate of HT.

### 4.4 Considerations on the observed associations in women

The associations of vitamins A and E with autoimmunity and thyroid function were observed specifically in our female cohort, which aligns with the marked female predominance of HT. The potential role of sex hormones, particularly estrogens, offers a plausible biological framework for these findings. Estrogens are known to promot humoral immunity and B-cell activation, which could lower the threshold for autoimmune reactivity (45, 46). Furthermore, sex hormones may influence the metabolism and distribution of fat-soluble vitamins. For instance, estrogen can modulate genes involved in vitamin A metabolism (47), and higher fat mass in women may alter the storage of lipophilic vitamins like E (48). Thus, an estrogen-enriched microenvironment could synergize with vitamin A and E deficiencies to exacerbate the autoimmune response in HT, leading to the more robust associations observed in women. This hypothesis merits investigation in future studies measuring sex hormone levels alongside vitamin status.

### 4.5 Reverse causality and oxidative stress

Reverse causality must be considered as an alternative explanation for our findings. HT is characterized by chronic inflammation and elevated oxidative stress, which may increase the consumption of antioxidant vitamins A and E. Therefore, the lower vitamin levels observed across disease categories may be a consequence rather than a cause of the autoimmune process. In this scenario, low vitamin levels would serve as a biomarker of disease activity rather than a modifiable risk factor. This possibility tempers the interpretation of our findings and impacts their clinical implications: if reverse causality is the primary driver, replenishing these vitamins may not alter the underlying autoimmune pathology, though it could still improve overall antioxidant status.

### 4.6 Limitations

Several limitations should be acknowledged. First, the cross-sectional design precludes causal inference. Second, the small male cohort limits the generalizability of findings in men. Third, we did not adjust for key confounders, such as iodine status, menopausal status, dietary intake, supplement use, or ultraviolet exposure. Fourth, blood sampling was conducted only in winter. While this controlled for seasonal variation, it may disproportionately affect vitamin levels, particularly vitamin D (due to reduced sunlight) and vitamin E (due to potential seasonal changes in lipid metabolism). Although vitamin A levels is less subject to seasonal variation, dietary influences across seasons cannot be ruled out. Fifth, no formal *a priori* sample size calculation was performed, increasing the risk of Type II errors. Finally, the lack of adjustment for multiple comparisons necessitates cautious interpretation of nominal *p*-values.

## 5 Conclusion

This exploratory study identifies distinct associations of vitamins A and E with disease activity in women with HT. These findings, specifically the inverse association of vitamin A with TgAb and of vitamin E with thyroid autoimmunity and dysfunction, highlight the need for longitudinal and interventional studies to further explore the relationship between these vitamins and HT.

## Data Availability

The raw data supporting the conclusions of this article will be made available by the authors, without undue reservation.
