# Supplementary material for: Vitamin A and E status across the spectrum of Hashimoto’s thyroiditis in women: associations with autoimmunity and thyroid function
Source: Front Nutr. 2026 Feb 19;13:1701808. doi: 10.3389/fnut.2026.1701808 (PMC12960084; doi:10.3389/fnut.2026.1701808)
Supplement: Supplementary file 1 [file Table_1.docx]

| **Table S1. Descriptive Characteristics of the Male Cohort by Thyroid Status (n=60).** | | | |
| --- | --- | --- | --- |
| **Parameter** | **HC** | **E-HT** | **HT-dys** |
| **n** | 35 | 21 | 4 |
| **Age(years)** | 49.60±9.94 | 47.62±11.21 | 44.25±11.98 |
| **Height(cm)** | 170.35±5.76 | 170.28±4.58 | 173.50±6.42 |
| **Weight(kg)** | 74.22±9.26 | 73.38±8.50 | 72.88±16.17 |
| **BMI(kg/m^2^)** | 25.55±2.61 | 25.32±2.90 | 24.01±3.82 |
| **SBP(mmHg)** | 132.23±11.57 | 127.14±10.98 | 124.50±12.04 |
| **DBP(mmHg)** | 82.23±9.42 | 77.62±10.05 | 73.50±11.24 |
| **FPG(mmol/L)** | 5.36±0.70 | 5.23±1.37 | 5.37±0.35 |
| **TG(mmol/L)** | 2.00±1.56 | 3.14±6.19 | 1.35±0.55 |
| **TCH(mmol/L)** | 5.12±1.32 | 5.11±1.15 | 5.48±1.34 |
| **LDL-C(mmol/L)** | 3.40±0.98 | 3.15±0.87 | 3.98±1.07 |
| **HDL-C(mmol/L)** | 1.14±0.23 | 1.20±0.24 | 1.32±0.16 |
| **UA(μmol/L)** | 450.74±90.35 | 430.39±88.22 | 448.40±116.70 |
| **Cr(μmol/L)** | 78.86±13.81 | 76.52±14.36 | 75.73±21.44 |
| **Urea(mmol/L)** | 5.05±2.30 | 5.03±0.91 | 5.09±0.69 |
| **ALT (U/L)** | 25.45±10.54 | 24.28±6.72 | 22.85±8.26 |
| **AST (U/L)** | 22.76±7.22 | 22.02±5.19 | 24.73±1.65 |
| **GGT (U/L)** | 28.13±12.01 | 31.07±9.10 | 28.18±13.51 |
| **FT4(pmol/L)** | 17.15±2.34 | 17.03±3.07 | 12.87±4.88 |
| **TSH(mIU/L)** | 1.67±0.96 | 3.07±1.62 | 21.09±18.04 |
| **TPOAb(IU/mL)** | 12.53±4.35 | 135.48±145.69 | 317.21±326.58 |
| **TgAb(IU/mL)** | 17.04±3.22 | 537.84±870.47 | 427.54±459.23 |
| **Vitamin A (ng/mL)** | 647.86±300.46 | 607.62±181.72 | 521.70±203.09 |
| **25(OH)D (ng/mL)** | 24.58±7.05 | 28.02±6.41 | 20.78±7.09 |
| **Vitamin D deficiency** | 0（0.00%) | 1 (4.76%) | 2 (50.00%) |
| **αT(ng/mL)** | 10264.52±3975.93 | 9914.36±3723.69 | 10197.33±4934.51 |
| **αT/(TCH+TG)(μg/mmol)** | 1442.23±304.13 | 1348.36±255.40 | 1416.00±392.26 |
| This table presents descriptive as mean ± SD or n (%) for the male cohort. Due to the very small sample size, particularly in the HT-dys group (n=4), no statistical comparisons were made. These data are presented for hypothesis-generating purposes only and should not be interpreted inferentially.  HT, Hashimoto's thyroiditis; HC, healthy controls; E-HT, euthyroid HT; HT-dys, dysfunctional HT; BMI, body mass index; SBP, systolic blood pressure; DBP, diastolic blood pressure; FPG, fasting plasma glucose; TG, triglycerides; TCH, total cholesterol; LDL-C, low-density lipoprotein cholesterol; HDL-C, high-density lipoprotein cholesterol; UA, uric acid; Cr, creatinine; ALT, alanine transaminase; AST, aspartate aminotransferase; GGT, γ-glutamyl transpeptidase; Cr, creatinine; FT4, free thyroxine; TSH, thyroid-simulating hormone; TPOAb, thyroid peroxidase antibody; TgAb, anti-thyroglobulin antibody; 25(OH)D, 25-hydroxyvitamin D; αT, α-tocopherol; αT/(TCH+TG), α-tocopherol/(total cholesterol+triglycerides). | | | |
