# Supplementary material for: Vitamin A and E status across the spectrum of Hashimoto’s thyroiditis in women: associations with autoimmunity and thyroid function
Source: Front Nutr. 2026 Feb 19;13:1701808. doi: 10.3389/fnut.2026.1701808 (PMC12960084; doi:10.3389/fnut.2026.1701808)
Supplement: Supplementary file 2 [file Table_2.docx]

| **Table S2*.* Exploratory analysis decomposing the statistical associations between αT/(TCH+TG), TPOAb, and thyroid function in women with Hashimoto's thyroiditis (n=82).** | | | |
| --- | --- | --- | --- |
| **Outcome Variable** | **Association Component** | **β (95% CI)** | ***p*** |
| TSH | Total association (αT/[TCH+TG] with TSH) | -0.0120 (-0.0210, -0.0030) | 0.009 |
|  | Association not explained by TPOAb | -0.0110 (-0.0190, -0.0020) | 0.015 |
| FT4 | Total association (αT/[TCH+TG] with FT4) | 0.0040 (0.0020, 0.0060) | 0.016 |
|  | Association not explained by TPOAb | 0.0002 (0.0001, 0.0004) | 0.025 |
| Note: This analysis presents a statistical decomposition of cross-sectional associations and does not imply causal mediation or temporal sequence.  αT, α-tocopherol; αT/(TCH+TG), α-tocopherol/(total cholesterol+triglycerides); TSH, thyroid-stimulating hormone; FT4, free thyroxine; TPOAb, thyroid peroxidase antibody. | | | |
